# A rare case of midgut malrotation leading to small bowel obstruction in an adult: a case report

**DOI:** 10.3389/fsurg.2025.1756065

**Published:** 2026-01-14

**Authors:** Tian Wang, Yichi Xu, Jiawen Xu, Chunhua Zhen

**Affiliations:** Gastrointestinal Surgery Department, Guihang Guiyang Hospital, Guiyang, China

**Keywords:** adult, intestinal malrotation, intestinal obstrucion, midgut malrotation, surgery

## Abstract

**Introduction:**

Small bowel obstruction is a common acute abdominal condition in adults, but obstruction caused by midgut malrotation—a congenital anomaly affecting the normal rotation of the intestine during fetal development—is rare. However, among infants and children, midgut malrotation is more frequently observed.

**Case Presentation:**

We report the case of a 40-year-old male patient who presented with vomiting, accompanied by cessation of passing flatus and defecation, along with mild abdominal pain. After emergency admission and an enhanced CT scan of the abdomen, a typical mesenteric whirlpool sign was observed, with the cecum and appendix located in the left lower abdomen. The patient underwent emergency surgery and recovered well postoperatively.

**Discussion:**

Midgut malrotation may be asymptomatic in the early stages but is accompanied by an increased risk of intestinal obstruction and intestinal necrosis with an increased angle of torsion, and early surgical intervention is recommended for midgut malrotation in adults.

**Conclusion:**

Midgut malrotation often leads to the development of intestinal obstruction in adults and is a rare cause. Imaging can be well defined, as well as determining the presence of critical conditions such as intestinal necrosis. Surgery is the only effective method of treatment at present, and early surgery after definitive diagnosis is very important.

## Introduction

Midgut malrotation is a congenital anomaly characterized by the abnormal rotation of the intestine during fetal development, which can lead to serious complications such as intestinal obstruction and necrosis. The foundational theory of midgut rotation, proposed by Mall and Frazer over a century ago, describes a 270 ° counterclockwise rotation around the superior mesenteric artery, occurring in three distinct stages from weeks 4 to 12 of gestation. During this process, the midgut descends into the abdominal cavity, and the mesentery fuses with the peritoneum, establishing normal intestinal positioning (1, 2). While midgut malrotation is predominantly diagnosed in neonates and infants, its presentation in adults is exceedingly rare, with an incidence rate of approximately 0.2% (3, 4). This rarity often results in delayed diagnosis and increased morbidity, as many adult patients may remain asymptomatic or present with nonspecific gastrointestinal symptoms such as abdominal pain and vomiting (5, 6).

The case presented herein involves a 40-year-old male who experienced vomiting, cessation of flatus and defecation, and mild abdominal pain, ultimately diagnosed with midgut malrotation leading to small bowel obstruction. This case is significant due to the rarity of adult presentations of midgut malrotation and highlights the importance of considering this condition in differential diagnoses for patients presenting with gastrointestinal symptoms, particularly when accompanied by signs of obstruction (7, 8). Early recognition and surgical intervention are crucial to prevent severe complications, including bowel ischemia and necrosis, which can arise from the increased angle of torsion associated with this condition (9).

## Case presentation

A 40-year-old man presented with vomiting and cessation of bowel movements. The vomiting was non-projectile and contained stomach contents. The patient was admitted to the hospital as an emergency due to mild abdominal distension that was not relieved by rest at home. After a thorough abdominal CT examination in the emergency department, the imaging suggested small bowel torsion and incomplete small bowel obstruction. The patient presented with abdominal distension and episodes of vomiting at the age of 8; after conservative treatment, the symptoms improved. To date, the patient has experienced similar episodes several times, all of which improved following conservative treatment. Overall, the patient's condition has responded well to conservative management.

On physical examination, the heart rate was 112 beats per minute, body temperature was 36.5 °C, blood pressure was 128/82 mmHg, and respiratory rate was 20 breaths per minute. The circulatory system was stable, with a stable heart rate and blood pressure. Abdominal examination revealed tenderness to pressure over the navel, with no signs of peritonitis. Digital rectal examination did not reveal any swelling or blood.

The patient's complete blood count showed a total leukocyte count of 10.83 × 10^9^/L, neutrophil percentage of 78.9%, neutrophil count of 8.35 × 10^9^/L, hemoglobin of 123 g/L, and platelets of 233 × 10^9^/L. Coagulation, liver, and kidney functions were normal, and serum potassium was 3.41 mmol/L.

**Figure 1:**
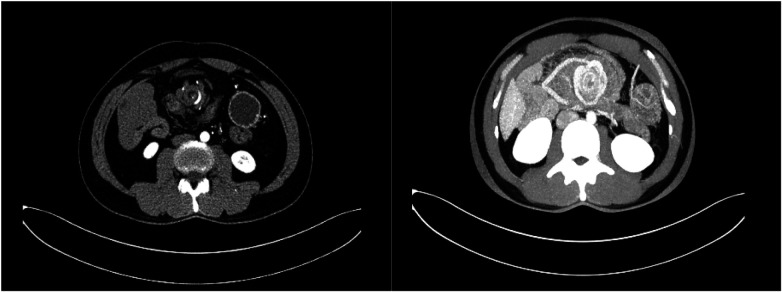
Swirling changes in the superior mesenteric artery are seen in the axial position.

**Figure 2:**
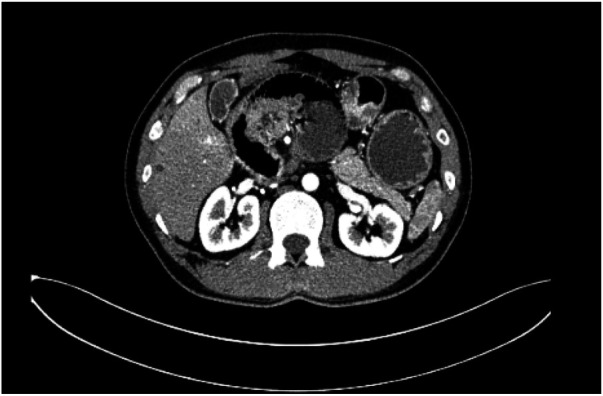
Axial view of the beginning of the jejunum and duodenum dilatation with pneumatosis and effusion.

Spiral-enhanced CT of the abdomen showed that the ileocecal bowel and appendix were located in the left lower abdomen. The duodenum and jejunum did not cross the midline, with the jejunum situated in the right upper abdomen. The superior mesenteric vein was located to the left of the superior mesenteric artery. A swirling pattern was observed involving the ascending and transverse colons, the duodenum, the jejunum, and the adjacent mesentery and blood vessels. The duodenum and part of the jejunum were dilated, contained fluid, and showed the presence of gas. A fluid-gas level was observed (Figures 1, 2).

**Figure 3:**
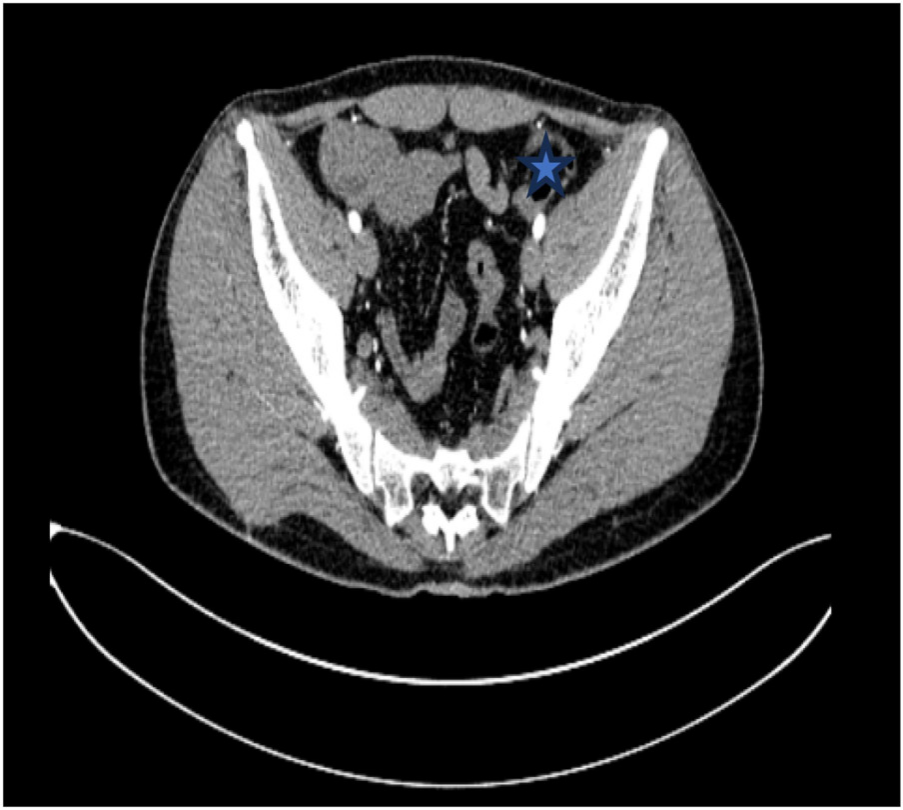
The labelled site is the appendix, which is located in the left lower abdomen.

**Figure 4:**
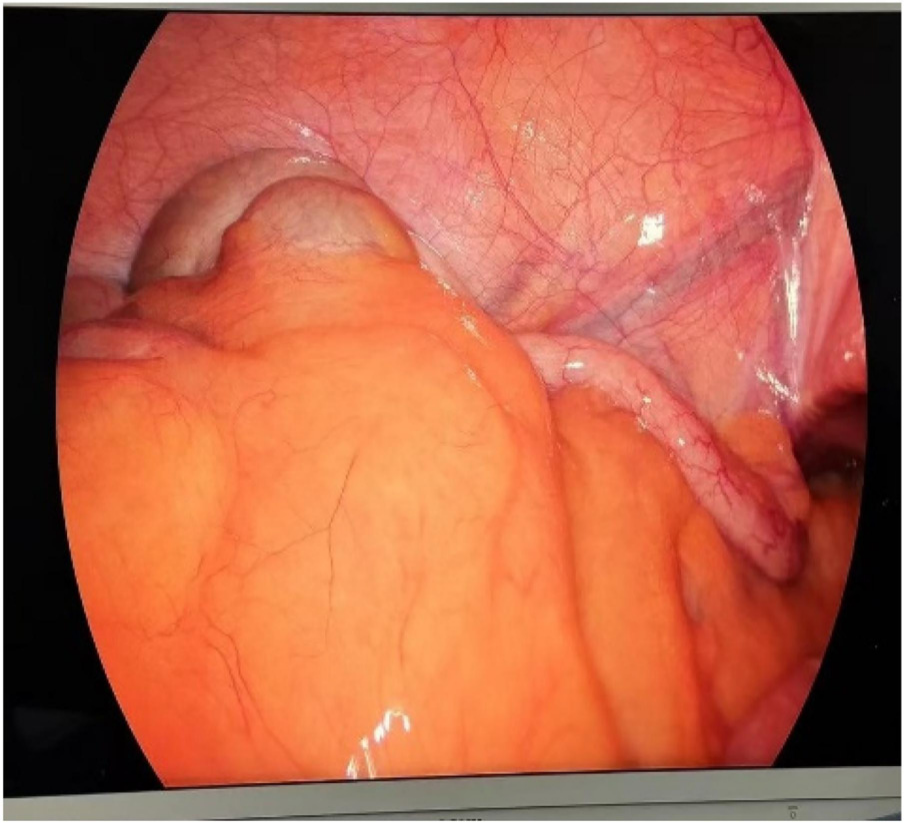
Intraoperative view of the cecum and appendix in the left iliac fossa.

After the patient was admitted to the hospital, he was treated with antispasmodic therapy, acid suppression therapy, and rehydration. Gastrointestinal decompression was also performed using a nasogastric tube. Based on the patient's enhanced abdominal CT results, we consider that the angle of intestinal torsion is large, making the possibility of spontaneous recovery low. This condition may lead to worsening symptoms, intestinal necrosis, or other complications. Laparoscopic exploration was performed in an emergency. The jejunum, transverse colon, and ascending colon were found to be rotated around the superior mesenteric vessels. Additionally, adhesions were detected between the mesentery and its root. The cecum and appendix are positioned in the left lower abdomen, an unusual anatomical location compared to their typical position in the right lower abdomen. (Figures 3, 4). Owing to the volvulus of the patient's bowel and the difficulty in performing detorsion laparoscopically, we decided to perform an open surgery.

**Figure 5:**
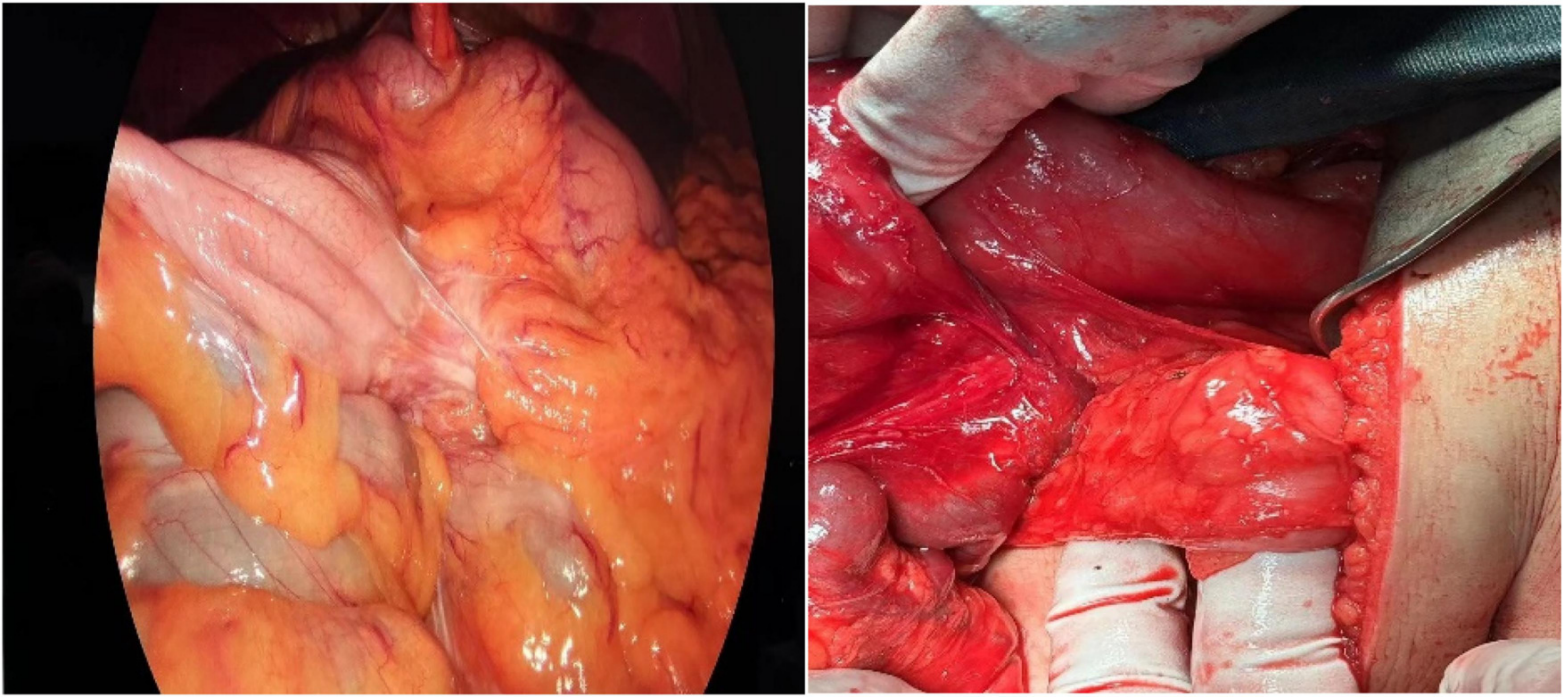
Intraoperative adhesion band seen between intestinal collaterals.

**Figure 6:**
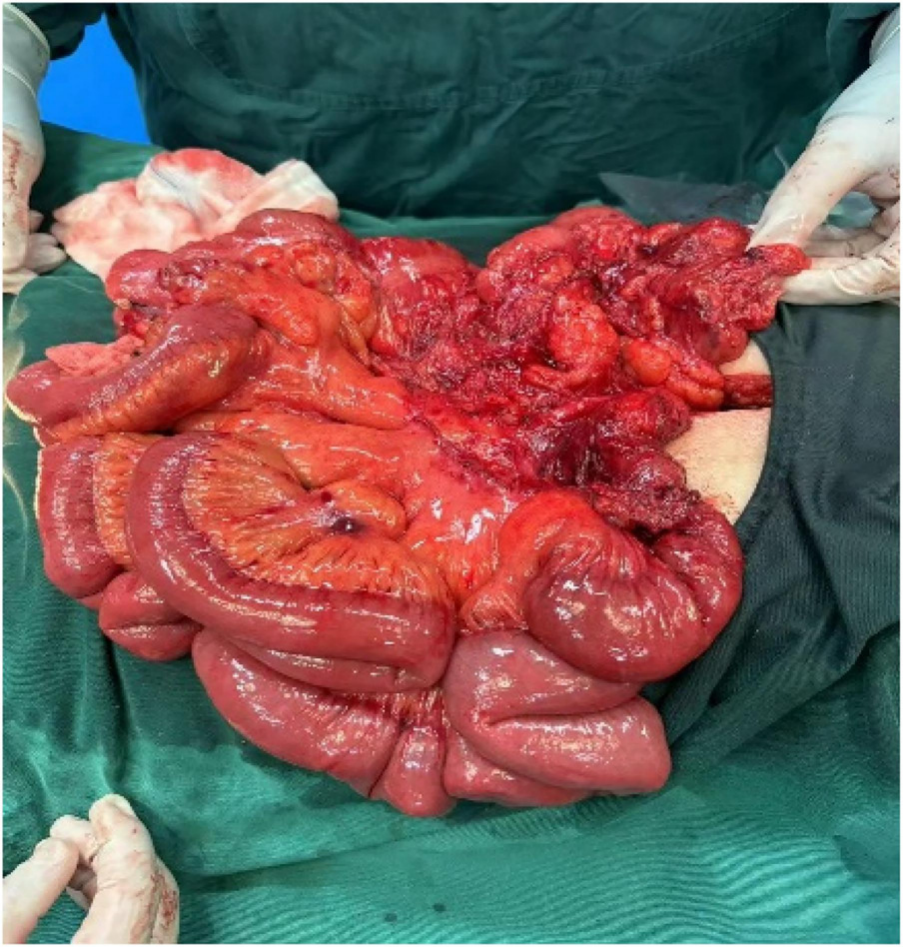
Intraoperative localised oedema of the small bowel was seen without necrosis.

An incision was made along the midline in the epigastric region, and the cecum and appendix were seen in the right lower abdomen. There were many adhesive bands between the intestinal collaterals (Figure 5). There was no necrosis of the bowel, only edema (Figure 6); the edema was improved by therapeutic heating. The adhesive bands between the cecum, abdominal wall, duodenum, and terminal ileum were then released to restore these anatomical structures to their normal positions. Then, we performed a Kareem procedure to repair intestinal malrotation, including duodenopexy to fix the ascending colon and cecum, as well as reconstruction of the Treitz Angle and mesenteric root (4). Subsequently, an appendectomy was performed. The patient was stable postoperatively and was discharged with a good recovery. The patient was followed up via telephone for six months, and during this period, had no symptoms of recurrent intestinal obstruction.

## Discussion

Midgut malrotation is primarily diagnosed in pediatric populations. The incidence of midgut malrotation in adults is low, and only sporadic case reports have been documented in the literature (5). In our reported case, we followed up the patient's medical history and found that the patient presented with abdominal pain when he was 8 years old, and the symptom had been with the patient for a long time. It was not until after consulting our hospital that the diagnosis was finally confirmed. Thankfully, the patient's bowel did not become necrotic.

As we described before, our digestive tract, in developing from the embryo, is divided into the foregut, the midgut, and the hindgut. And our midgut rotates most predominantly. It can be divided into three stages. Therefore, an abnormality at any of these stages can lead to malrotation. Intestinal malrotation was reported by Ladd in 1936 and defined as a congenital abnormality of intestinal rotation and fixation during fetal development (6).

There are three types of malrotation according to the trustee frame classification. Type 1: no rotation, type 2: duodenal malrotation, and type 3: duodenal plus cecum malrotation. In addition, malfixation of the mesentery and stenosis of the vascularised tip can lead to intestinal torsion (7).

In paediatric patients, intestinal malrotation presents early with symptoms of small bowel obstruction (abdominal distension and bilious vomiting), which can be diagnosed by upper gastrointestinal imaging series. Many adult patients may be asymptomatic or have only nonspecific gastrointestinal symptoms such as abdominal pain and vomiting. Malrotation in adults is a rare cause of intestinal obstruction with a necropsy rate of 1/6,000 (8).

The reported incidence in newborns is 1/500, but the incidence of symptomatic malrotation in newborns is about 1/6,000. The most common symptoms in newborns are abdominal distension and bilious vomiting. Torsion or intestinal obstruction usually occurs in 64- 80% of patients within the first month of life. Adult presentation is extremely rare, with an incidence of 0.2% (9). As a result, diagnosis is usually delayed, morbidity is significantly increased, and some patients may remain asymptomatic throughout their lives or develop symptoms insidiously during postprandial periods in adulthood, such as intermittent bilious vomiting (30%), intermittent abdominal pain (20%), dyspareunia, oral intolerance, chronic diarrhoea, and malabsorption (6).

Adult midgut malrotation is rare, and multislice spiral CT can be used to diagnose and classify it in adults. The pattern of adult midgut malrotation may be excellent. The detection of typical intestinal malposition, extra-intestinal abnormalities (especially pancreatic leptomeningeal hypoplasia), and SMV/ SMA orientation abnormalities may lead to a correct diagnosis (10).

In the case we report, the typical swirl sign, as well as the cecum and appendix hovering in the left lower abdomen, were noted on preoperative CT. Intraoperatively, a more visualised presentation consistent with CT was observed. The ligamentum radiatum was also identified. Midgut malrotation is mostly diagnosed by imaging. There are also reports in the literature that, for example, barium enema, CT, or MRI may be useful but are not routinely used (4). However, the authors disagree and believe that abdominal CT is of great value in the diagnosis of the disease, on the one hand, to clarify intestinal torsion and to exclude other acute abdominal conditions, and on the other hand, to provide an important basis for surgery.

The treatment of intestinal malrotation and its complications is surgical. The procedure of choice is Ladd's procedure (11), which was first described in the 1930s. It consists of untwisting the small bowel in the presence of small bowel torsion, separating adhesions (ladd's bands), compressing the duodenum, enlarging the mesentery to prevent recurrence of intestinal torsion, and placing the bowel in a nonrotational position, with the small bowel on the right side of the abdomen and the colon on the left side. Laparoscopic surgery can also be attempted nowadays with the development of laparoscopic techniques, but it is limited to selected patients (4). Laparoscopic surgery has a shorter hospital stay than caesarean section. However, in emergencies such as intestinal necrosis or midgut torsion, caesarean section should be chosen (12).

In 2021, Kareem Abu-Elmagd published a new technique for the treatment of malrotation called malrotation repair surgery or Kareem's procedure (4). Which is specific in that instead of placing the bowel in a non-rotational position, it repairs malrotation by placing the bowel in a normal rotational position. One of the main advantages of this technique over Ladd's approach is that there is theoretically no risk of torsion when repairing malrotation, and significant improvement in gastrointestinal symptoms was seen before and after the procedure, as assessed by the NIH-PROMIS scale, as well as in 34 patients who initially underwent Ladd's procedure and then went on to undergo Kareem's procedure (4).

## Conclusions

This report emphasises the importance of including intestinal malrotation as one of the differential diagnoses in adults presenting with gastrointestinal symptoms. Although intestinal malrotation is usually associated with pediatric patients, it can manifest in adulthood and exhibit a variety of clinical features that make the diagnosis challenging. Early recognition of intestinal malrotation is essential to prevent potentially serious complications (e.g., intestinal volvulus or intestinal ischemia). Consequently, surgical intervention remains the mainstay of treatment for symptomatic intestinal malrotation in adults. Timely surgical correction reduces pain, prevents complications, and improves the patient's quality of life. Overall, this report suggests that intestinal malrotation should be considered in the differential diagnosis of adults presenting with gastrointestinal symptoms. Moreover, abdominal CT plays a crucial role in diagnosing intestinal malrotation to ensure accurate diagnosis and optimal patient management.

## Data Availability

The original contributions presented in the study are included in the article/Supplementary Material, further inquiries can be directed to the corresponding author/s.

## References

[1] Frazer JE Robbins RH. On the factors concerned in causing rotation of the intestine in man. J Anat Physiol. (1915) 50(Pt 1):75–110. 17233053 PMC1289077

[2] Soffers JH Hikspoors JP Mekonen HK Koehler SE Lamers WH. The growth pattern of the human intestine and its mesentery. BMC Dev Biol. (2015) 15(1):31. 10.1186/s12861-015-0081-x 26297675 PMC4546136

[3] Wang Y Gu Y Ma D Guo W-X Zhang Y-F. Congenital intestinal malrotation with gastric wall defects causing extensive gut necrosis and short gut syndrome: a case report. World J Clin Cases. (2022) 10(9):2851–7. 10.12998/wjcc.v10.i9.2851 35434107 PMC8968819

[4] Ibarra Rodríguez I Gavilanes Salazar G Ruiz Jiménez I Sáenz Dorado A Charmorro Juárez MR Bueno Recio RJ. A new technique in the treatment of intestinal malrotation. Cir Pediátr. (2023) 36(4):191–4. 10.54847/cp.2023.04.16 37818902

[5] Yin MD Hao LL Li G Li Y-T Xu B-L Chen X-R. Adult-onset congenital intestinal malrotation: a case report and literature review. Medicine (Baltimore). (2024) 103(8):e37249. 10.1097/MD.0000000000037249 38394530 PMC11309662

[6] Perez Galaz F Moedano Rico K Pérez Tristán FA Acuña Macouzet A Jafif Cojab M. Midgut volvulus caused by intestinal malrotation; A rare cause of acute abdomen in adults. Case report. Int J Surg Case Rep. (2020) 73:355–9. 10.1016/j.ijscr.2020.07.051 32745727 PMC7398895

[7] Martinez SA Fligor SC Tsikis S Short M Corcoran KE Rogers A IMPOWER: a national patient-generated registry for intestinal malrotation exploring diagnosis, treatment, and surgical outcomes. Orphanet J Rare Dis. (2023) 18(1):113. 10.1186/s13023-023-02722-5 37170358 PMC10176693

[8] Eltayb AB Hegazi A Elhag O Abdelgadir A. Midgut volvulus due to congenital malrotation in an adult: a case report. J Med Case Rep. (2023) 17(1):378. 10.1186/s13256-023-04096-5 37620962 PMC10463942

[9] Ahmadi Amoli H Rahimpour E Firoozeh N Abbaszadeh-Kasbi A Jazaeri SA. Midgut volvulus is a rare cause of intestinal obstruction in adults: a case report. Int J Surg Case Rep. (2019) 58:41–4. 10.1016/j.ijscr.2019.03.029 31003093 PMC6475719

[10] Yang B Chen WH Zhang XF Luo Z-R. Adult midgut malrotation: multi-detector computed tomography (MDCT) findings of 14 cases. Jpn J Radiol. (2013) 31(5):328–35. 10.1007/s11604-013-0194-8 23475600

[11] Brady JT Kendrick DE Barksdale EM Reynolds HL. The Ladd procedure for adult malrotation with Volvulus. Dis Colon Rectum. (2018) 61(3):410. 10.1097/DCR.0000000000000998 29377870

[12] Mizuta N Kikuchi T Fukuda Y. Adult intestinal malrotation treated with laparoscopic Ladd procedure. Case Rep Surg. (2022) 2022:1–6. 10.1155/2022/6874885 PMC9596249 36304201

